# Metal Preferences and Metallation*

**DOI:** 10.1074/jbc.R114.588145

**Published:** 2014-08-26

**Authors:** Andrew W. Foster, Deenah Osman, Nigel J. Robinson

**Affiliations:** From the Department of Chemistry and School of Biological and Biomedical Sciences, Durham University, Durham DH1 3LE, United Kingdom

**Keywords:** Copper, Iron, Manganese, Nickel, Zinc, Irving-Williams Series, Metal Sensors, Metallochaperone, Metalloenzymes, Polydisperse Buffer

## Abstract

The metal binding preferences of most metalloproteins do not match their metal requirements. Thus, metallation of an estimated 30% of metalloenzymes is aided by metal delivery systems, with ∼25% acquiring preassembled metal cofactors. The remaining ∼70% are presumed to compete for metals from buffered metal pools. Metallation is further aided by maintaining the relative concentrations of these pools as an inverse function of the stabilities of the respective metal complexes. For example, magnesium enzymes always prefer to bind zinc, and these metals dominate the metalloenzymes without metal delivery systems. Therefore, the buffered concentration of zinc is held at least a million-fold below magnesium inside most cells.

## Introduction

This narrative sets out, with examples, how cells assist metallation. Such assistance is vital because the physical and chemical properties of proteins tend to select essential divalent metal ions with a ranked order of preference that follows the Irving-Williams series (1).

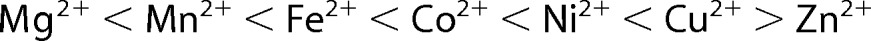
 Competitive metals must be kept out of binding sites for the weaker binding ions. Cupric ions are at the top of the series, although their order with respect to zinc can flip (2). In the reducing conditions of the cytoplasm, cuprous (Cu^+^) rather than cupric (Cu^2+^) ions are expected to predominate, but these ions can also form tight complexes, especially with sites that contain sulfur ligands (3). In the periplasm of bacterial cells, ferric (Fe^3+^) rather than ferrous (Fe^2+^) ions often dominate (4). Ferric ions are retained in solution in organic complexes that can be exceptionally tight and include binding proteins such as the ferric-binding protein (Fbp) in the bacterial periplasm (5).

Because proteins are not rigid, the scope for steric selection of metal cofactors is imperfect. Mismetallation can exploit a subset of ligands and/or distort the native binding geometry. Typically, a protein becomes inactive if one or more residues of an active metal site are recruited to an alternative site, perhaps with alternative geometry, by a more competitive metal. For example, glyoxalase of *Clostridium acetobutylicum* (GlxI) is activated by nickel or cobalt, both of which assume octahedral geometries, whereas zinc binds tightly in trigonal bipyramidal geometry and inactivates this isoform of the enzyme (6).

**FIGURE 1.**
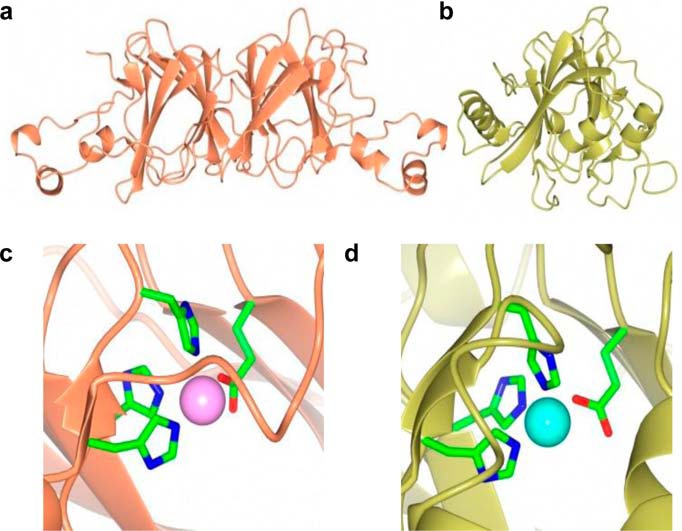
**Metallation is governed by metal availability for MncA and CucA.** *a*, Mn(II)-MncA global fold. *b*, Cu(II)-CucA global fold. Both proteins adopt a cupin architecture, with MncA composed of two cupin domains. *c*, MncA N-terminal Mn(II)-binding site. *d*, CucA Cu(II)-binding site. Both proteins coordinate their metals with identical ligand sets, with a water molecule in the open coordination position (this position is occupied by acetate in the C-terminal Mn(II)-binding site of MncA). MncA and CucA both prefer to bind copper rather than manganese *in vitro*, but MncA folds and traps manganese in the metal-regulated environment of the cytoplasm. Protein Data Bank (PDB) codes: 2VQA and 2XL7.

Correct metallation *in vivo* is favored because the cytoplasm is a metal-controlled environment. For example, two periplasmic cupins (manganese MncA and cupric CucA) from a model cyanobacterium bind metal via analogous ligand sets within analogous folds (Fig. 1), yet *in vivo* they acquire different metals. MncA and CucA both show *in vitro* metal preferences that match the Irving-Williams series, which is especially problematic for MncA. A 10,000× and 100,000× excess of manganese is required at MncA folding in order for manganese to outcompete cupric or zinc ions, respectively (7). Cuprous ions can also outcompete manganese. Manganese MncA has oxalate decarboxylase activity, whereas neither the zinc nor the copper forms are active (7). CucA is a Sec substrate that folds in the periplasm on secretion, whereas MncA is a Tat substrate. The Tat system translocates prefolded proteins, and hence MncA folds within the cytoplasm before export (7, 8). In this way, MncA entraps manganese before exposure to copper and zinc in the periplasm. In the cytoplasm, at the site of MncA folding, copper and zinc must be at least 10,000 and 100,000× less available than manganese. This must reflect the relative buffered concentrations of these three metals plus, hypothetically, a manganese delivery system for MncA.

## When Metals Compete with Other Metals for Proteins

Metal availability within cells is restricted such that proteins compete with other molecules, including other proteins, for limited pools of the most competitive metals. Dudev and Lim (9) have assessed the physical and chemical properties of metals and proteins that influence metal preferences. These include valence, ionic radius, coordination geometry, ligand number, second-shell ligands, effects of the protein matrix, and ligand characteristics (net charge, dipole moment and polarizability, charge-donating/-accepting ability, and denticity) (9). Despite these opportunities to tune metal preferences, *in vitro* metallation is typically aberrant when essential metals simply compete with each other for proteins (7).

Zinc and magnesium are the most commonly utilized metal cofactors (∼16 and ∼9% of all enzymes, respectively) (10), and they dominate the subset of metalloenzymes lacking a defined delivery system, representing ∼78% of this group (Table 1). Empirically, zinc is known to replace magnesium to inactivate enzymes including β-galactosidase (11), tyrosine kinases (12), and magnesium alkaline phosphatase (13, 14). The calculated free energies for replacing magnesium with zinc in rigid or flexible sites implies that zinc will always be favored over magnesium in mono- and binuclear binding pockets, with Δ*G* for replacement in flexible, neutral sites ranging from −10 to −29 kcal mol^−1^ (15). The incorporation of magnesium into chlorophyll to metallate chlorophyll-binding proteins is a special case that exploits delivery systems and is therefore considered separately in a later section of this minireview.

**TABLE 1:** **Types of metal sites and metal delivery pathways in Metal MACiE**

Metal and site type	Example enzyme from Metal MACiE*^a^*	Delivery pathway/chaperone	% of Metal MACiE total*^b,c^*
**Magnesium**			
Mononuclear	Adenylate cyclase (M0058)	None known	38
Trinuclear (magnesium)	Trichodiene synthase (M0262)	None known	3

**Manganese**			
Mononuclear	Xylose isomerase (M0308)	None known	8
Trinuclear (manganese or zinc)	Deoxyribonuclease IV (M0011)	None known	<1

**Iron**			
Mononuclear	Catechol 2,3-dioxygenase (M0034)	None known	3
Dinuclear (FeFe)	Ferredoxin hydrogenase (M0127)	HydE/G provide iron as [FeS], production of which is dependent on CyaY	<1
Dinuclear (NiFe)	Cytochrome *c*3 hydrogenase (M0126)	Assembly of cyano-, carbonyl-coordinated iron occurs on HypD; source of iron is unknown	<1
Dinuclear (ZnFe)	Purple acid phosphatase (M0043)	None known	<1
Heme	Ubiquinol-cytochrome *c* reductase (M0208)	Iron chelatase	7
Iron-sulfur cluster	Aldehyde oxidase (M0105)	CyaY	14

**Cobalt**			
Mononuclear	Thiocyanate hydrolase (M0284)	None known	2
Cobalmin	Methionine synthase (M0268)	CbiX	2

**Nickel**			
Dinuclear (NiFe)	Cytochrome *c*3 hydrogenase (M0126)	HypA/ HypB/ SlyD	<1
Dinuclear (NiNi)	Urease (M0087)	UreE/ UreG	<1
Factor-430	Coenzyme B sulfoethylthiotransferase (M0156)	None known	<1

**Copper**			
Mononuclear	Copper-zinc SOD (M0138)	CCS (and others)	2*^d^*; 1*^e^*
Dinuclear (CuCu)	Tyrosinase (M0125)	Atx1 (and others)	1
Dinuclear (CuMo)	Carbon-monoxide dehydrogenase (M0107)	None known	<1

**Zinc**			
Mononuclear	Alcohol dehydrogenase (M0256)	None known	11
Dinuclear (ZnZn)	Beta lactamase (M0015)	None known	2
Dinuclear (ZnFe)	Purple acid phosphatase (M0043)	None known	<1
Trinuclear (zinc)	Phospholipase C (M0027)	None known	1

**Molybdenum**			
Molybdopterin	Xanthine dehydrogenase (M0139)	MoeA	2
FeMo cofactor	Nitrogenase (M0212)	CyaY, NifH	<1
Dinuclear (CuMo)	Carbon-monoxide dehydrogenase (M0107)	None known	<1

*^a^* Metal MACiE identifier shown in parentheses.

*^b^* Total excludes calcium enzymes represented in Metal MACiE.

*^c^* Hetero-dinuclear sites count as one site for each metal ion, and homo di- and tri-nuclear sites count as one site.

*^d^* Known delivery pathways.

*^e^* Unknown delivery pathways.

Iron and manganese are the next most common cofactors estimated to be exploited by ∼8 and ∼6% of enzymes (10). These ions account for most (∼18%) of the remaining fraction of metalloenzymes that are devoid of delivery systems, noting that another subset of iron enzymes does have metal delivery systems and iron is commonly found in preassembled cofactors. The divalent ions of manganese and iron have similar ligand affinities, radii, coordination preferences, and solvation free energies, creating a distinct challenge for proteins to discern between these elements when they compete for a site (9).

## Uncertain Metallation *in Vivo* and Cambialistic Proteins

With a few pioneering exceptions (16, 17), the extent of mismetallation *in vivo* is unknown. Current methods for native metalloproteomics are neither global nor high throughput (7, 18), and so the extent of post-translational regulation through metallation is unclear. The picture is further complicated because multiple metals support catalysis in so-called cambialistic enzymes. Acireductone dioxygenase (ARD)^2^ from *Klebsiella oxytoca* is currently a rare example of an enzyme that can catalyze two different reactions dependent upon metal occupancy (19). Iron·ARD is widespread, and the nickel·ARD-dependent pathway has been observed in *Bacillus subtilis* and *Escherichia coli*, but both forms have been recovered from *K. oxytoca*. However, there is currently no evidence that both forms of the enzyme confer a selective advantage to *K. oxytoca.* Fractional occupancies of ARD with nickel and iron remain to be investigated *in vivo*, as does the tantalizing possibility that metallation is switched to match metabolic need.

## Conformationally Trapped Metals and Opportunities for Proofreading of Metallation

There is scope for mismetallated proteins to be selectively degraded or recycled or to remain in a partially unfolded state. A subset of metal cofactors becomes kinetically trapped in proteins. The correct geometry can stabilize the fold, offering, in effect, the potential for proofreading of metal occupancy based upon second coordination shell interactions. For example, manganese in the copper·cupin CucA is readily replaced upon incubation with copper, but in the structurally related manganese·cupin MncA, manganese becomes trapped at folding and refractory to subsequent replacement by copper (7). Thus, folding and metal trapping are uncoupled from manganese binding to CucA, where this is mismetallation, but coupled to manganese binding in MncA. To date, *in vitro* biochemical studies of metal binding preferences of proteins have not included protein folding chaperones such as Hsp70 or its co-chaperones and nucleotide exchange factors. Association of chaperones with exposed hydrophobic patches of nascent proteins impacts upon the energetics of protein folding (20), but it remains to be tested whether or not this sometimes imposes a bias in favor of the correct metal.

## Metal Delivery Pathways

Fidelity in metallation with two competitive metals, nickel and copper, is typically assisted by metallochaperones (21–23). The term “metallochaperone” describes a collection of proteins, for a diversity of metals, which differ in their biochemical mechanisms. Known nickel chaperones, which include HypB, interact with a battery of other proteins with consumption of nucleotide cofactors aiding metal insertion (21, 22). When *Helicobacter pylori* HypB aberrantly binds zinc its GTPase, activity is not triggered, and in this way, cofactor delivery becomes selective for nickel (24). Known copper chaperones do not require nucleotide cofactors. Both copper and nickel chaperones introduce a kinetic bias into the partitioning of metals by engaging in specific protein-protein interactions that recognize the correct partners (23). Such interactions also orientate the donor and acceptor ligands to encourage facile ligand exchange (25).

Preassembled complex metal cofactors include cobalamin (cobalt), iron-sulfur clusters, heme and siroheme (iron), molybdopterin (molybdenum), F430 (nickel), and chlorophyll (magnesium). Discrimination between these more elaborate molecular assemblies as opposed to individual metal ions at cofactor selection is less challenging, but nonetheless may be aided by delivery proteins. For example, monothiol glutaredoxins (Grxs) and BolA proteins play roles in [FeS] cluster delivery as well as iron sensing (26), with yeast strains deficient in Grx3 and Grx4 exhibiting defects in multiple iron-dependent enzymes (27, 28); NarJ assists in the insertion of molybdopterin into nitrate reductase in *E. coli* cells (29), and CcmE functions as a heme chaperone in the periplasm of *E. coli*, delivering its cargo to CcmF for insertion into cytochrome *c* (30).

Metallochaperones that contribute toward fidelity in partitioning metals during complex cofactor assembly include chelatases for heme, cobalamin, and chlorophyll (31, 32) and MoeA for molybdopterin (33). Ferrochelatases, for example, can catalyze the insertion of metals other than iron into tetrapyrroles, such that zinc protoporphyrin IX becomes diagnostic for some iron deficiencies (34). The metal preferences and metallation of metallochaperones warrant investigation.

The majority of copper proteins are secreted, and copper efflux from the cytosol is driven by P_1_-type ATPases that acquire copper from metallochaperones such as Atx1 (35, 36). Exactly how copper is then handed to nascent proteins post-secretion is the topic of current investigations. Oddly, CucA in the cyanobacterial periplasm has impaired metallation in mutants missing copper-transporting P_1_-type ATPases (CtaA and PacS), and the mutant periplasm is devoid of CucA but enriched with low *M*_r_ copper complexes (37). Thus, copper is routed via the cytoplasm and the cyanobacterial copper chaperone Atx1, before export via a P_1_-type ATPase to load CucA. Moreover, secretion of CucA seems to be coupled to copper efflux (37). A subset of P_1_-type ATPases that have tight *K_m_* and low *V*_max_ does not confer copper resistance but appears to support metal delivery to nascent cupro-proteins (38). There is evidence of interaction between *E. coli* periplasmic copper chaperone CusF and P_1_-type ATPase CopA, whereas periplasmic copper chaperone CueP is required for metallation of SodCII in *Salmonella enterica* sv. *Typhimurium* (39, 40).

## Evaluating the Contribution of Delivery Pathways to Metallation

To estimate the fractions of metalloproteins that bind preassembled cofactors or are otherwise metallated via metallochaperones, the Metal MACiE database has been interrogated. Metal MACiE is a manually curated catalogue of enzymes that require metals for their catalytic mechanisms and for which a protein structure has been determined (41). Metal ions solely performing structural roles in proteins that are not enzymes are not annotated in Metal MACiE. This is liable to lead to an under-representation of zinc, which is widely used in zinc fingers (42). With such limitations in mind, Metal MACiE can be used to make first approximations of the proportions of enzymes with various metal centers. Table 1 lists the types of sites in the database, noting where proteins are known to assist in metal delivery directly to the enzyme (exemplified by nickel and copper), to a subcellular compartment containing the enzyme (exemplified by copper in the secretory system or periplasm), or to preformed metal cofactors. In total, 30% of metalloenzymes within the database are estimated to lie at the end of such delivery pathways, and metalloenzymes are estimated to account for almost half of all enzymes (43).

It is uncertain where most metallochaperones acquire metal and to what extent their relative metal affinities correspond to the metal requirements of the delivery pathways. Cyanobacteria are useful models for exploring partitioning among metallochaperones. In common with other photosynthetic organisms, they have a high demand for metals (44), but they also have delivery proteins for an especially wide range of metals: Atx1 for copper to thylakoids (45), UreE and HypA/B for nickel to urease and hydrogenase (46), ferrochelatase for iron to heme and siroheme (47), magnesium chelatase for magnesium to chlorophyll (48), CbiX for cobalt to cobalamin (plants in contrast do not make cobalamin) (49), MoeA for molybdenum to molybdopterin, CyaY for iron to iron-sulfur clusters, and possibly PratA for manganese to photosystem II (50). A set of metal competition experiments between the purified cyanobacterial metallochaperones could establish whether or not their relative metal affinities simply enable metals to partition to the correct delivery pathway. This in turn would resolve the metallation challenge for ∼30% of metalloenzymes.

Alternatively, metallochaperones might directly acquire metal from importers assisted by specific protein interactions. The idea that inward metal transport is coupled to the loading of delivery pathways, to channel metals to sites of metalloenzyme assembly, is widely envisioned but sparsely evidenced. Notably, analyses of yeast mutants did not identify any single copper donor for either of two copper metallochaperones (51). Nonetheless, there is evidence that the copper chaperone for superoxide dismutase (CCS) can interact with membranes and with the copper importer Ctr1 (52), and metal transfer to Atx1 has also been observed *in vitro* using a cytosolic domain of Ctr1 (53). Nickel imported by the Nik system is destined for hydrogenase and largely unavailable to nickel-responsive transcriptional regulators (54), which might also suggest direct handover of nickel to HypA/B. However, evidence that the substrate for the Nik importer is a nickel-histidine complex provides an alternative explanation for these observations if HypA/B can preferentially acquire nickel from nickel-histidine (55). There is evidence that a mitochondrial iron importer mitoferrin-1 interacts with a ferrochelatase for heme biogenesis (56). This iron supply pathway cannot be “hardwired” exclusively for iron if zinc protoporphyrin IX accumulates under iron deficiency (34). Iron-sulfur clusters are the targets for surplus cobalt and copper (57–60). Both cobalt and copper directly destabilize the assembled cluster on the scaffold proteins and, at least for cobalt, it is known that the resultant mixed cluster can be delivered to apo-proteins (58, 59). Thus, imperfect metal preferences of delivery systems can sometimes propagate mismetallation.

Metallochaperone-catalyzed delivery of the more competitive metals, such as nickel and copper, enables cells to more efficiently cofactor a subset of proteins with these ions. However, viewed from a different perspective, such metal delivery supports metallation at low buffered concentrations sufficient to exclude these elements from binding sites for metals lower down the Irving-Williams series (1). For example, cyanobacterial mutants missing the copper metallochaperone Atx1 show phenotypes indicative of the mismetallation of binding sites for other metals with copper (61).

## The Set Points for Metal Homeostasis

**FIGURE 2.**
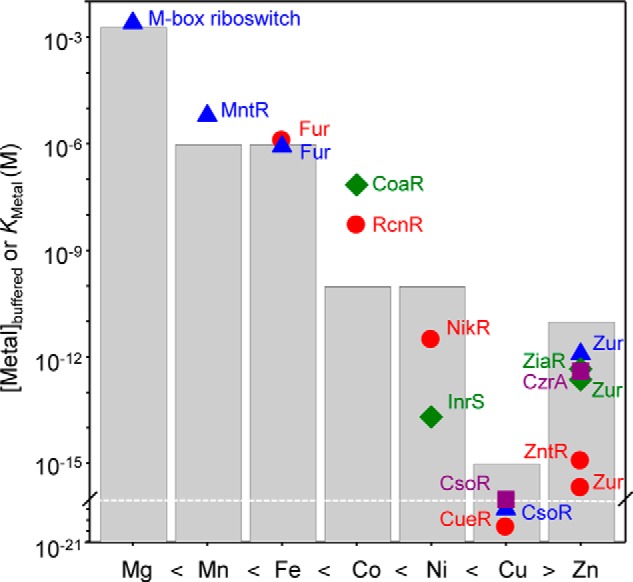
**Correlation between buffered set points and metal sensor affinities.** Shown are graphical representations of estimated intracellular buffered metal concentrations (*gray bars*) for magnesium, manganese, iron, cobalt, nickel, copper, and zinc (62, 65, 66, 72, 73, 90, 91) and correlation with *K*_Metal_ of cytosolic metal sensors for their cognate metal, including Fur (92), RcnR (93), NikR (94), CueR (89), Zur (88), and ZntR (88), from *E. coli* (*red circles*); the M-box riboswitch (95), MntR (96), Fur (96), CsoR (97), and Zur (98), from *B. subtilis* (*blue triangles*); CoaR (84), InrS (83), Zur (85), and ZiaR (85) from *Synechocystis* PCC 6803 (*green diamonds*); and CsoR (99) and CzrA (100) from *Staphylococcus aureus* (*purple squares*). It is hypothesized that *K*_Metal_ of metal sensors maintains the set points for buffered metal concentrations as an inverse function of the Irving-Williams series.

The buffered (rather than total) set points for metals can vary between cell types and intracellular compartments and throughout the lifetime of a cell. Nonetheless, magnesium appears to be universally held at ∼10^−3^ m inside cells (Fig. 2, *gray bars*), about 10 times less than the concentration in sea water and 10 times more than typical concentrations in fresh water (62, 63). Proteins that require ferrous ions often exhibit affinities of ∼10^−7^ m, which is suggested to match the ferrous concentration in the sulfide-rich anaerobic conditions when life first evolved (64). By determining the ferrous affinity of glutathione (glutathione has a concentration of ∼2–10 mm within the cytoplasm), and assuming that this complex is a major component of the cytosolic iron pool, a value in the region of 10^−6^ to 10^−7^ m for the buffered concentration of ferrous iron is plausible (65) (Fig. 2, *gray bars*).

The cytosolic concentration of manganese has been estimated to be comparable with ferrous iron (66, 67) (Fig. 2, *gray bars*). However, manganese concentrations may be elevated within organelles such as the chloroplast or mitochondria where there is high demand. In a bacterial cytosol, the concentration of manganese can vary. For example, in response to oxidants, manganese is elevated to correctly metallate manganese superoxide dismutase (SOD) (16). Nickel- and cobalt-requiring enzymes are thought to have been more prevalent in early anaerobic life, and Fraústo da Silva and Williams (62) suggest that these two metals are unlikely to have ever exceeded 10^−10^ m in the cytosol (Fig. 2, *gray bars*).

Zinc-binding sites in most proteins have affinities that are typically 10^−11^ m or tighter (68). The use of either synthetic or genetically encoded zinc-responsive fluorophores has placed buffered zinc concentrations within the cytosol of bacteria and eukaryotic cells in the 10^−12^ to 10^−10^ m range (69–72). Buffered cytosolic copper concentrations have been estimated to be ∼10^−15^ m or less using copper-responsive fluorophores (73, 74) (Fig. 2, *gray bars*). In yeast, copper zinc SOD1 has a copper affinity of 10^−15^ m but requires the CCS metallochaperone for activation *in vivo*. Consequently, it was inferred that copper must be buffered below 10^−15^ m (75). CCS additionally catalyzes the formation of a vital disulfide bond within SOD1 (76), providing an alternative explanation for inactivity of SOD1 in CCS-deficient cells.

What sustains these different buffered metal concentrations? An expectation is that this relates to detection thresholds of sensors that control homeostasis for the respective metals. There are pitfalls in the estimation of *K*_Metal_, especially for tighter binding elements (77), generating a jumble of erroneous values. Nonetheless, mindful of this caveat, a remarkable correlation exists between estimates of *K*_Metal_ for metal sensors and estimates for buffered cytosolic metal concentrations (Fig. 2). This observation is consistent with the intracellular set point for metal homeostasis being a function of these sensor affinities. By setting the metal affinities of metal sensors such that those for the most competitive metals are the tightest, the control of metal efflux, metal influx, and metal sequestration and the switching of metabolism to spare limiting metals are thus primed to maintain the buffered metal concentrations as an inverse function of the Irving-Williams series. Under this regime, subtle differences in the relative metal preferences of metalloenzymes now become sufficient to enable correct *in vivo* metallation.

## How a Cell's Set of Metal Sensors Acts in Concert to Discern Metals One from Another

The actions of metal sensors help maintain buffered metal concentrations, and these concentrations in turn influence which metals are acquired by ∼70% of metalloenzymes. Thus, the metal specificity of metal sensors becomes a dominant factor in the fidelity of metallation. The proportion also becomes even higher than 70% if some metallochaperones are metallated from buffered metal pools. Metal-sensing, DNA-binding transcriptional regulators have been extensively characterized in bacteria (78, 79) and identified for copper, iron, and zinc in yeast (80, 81). However, where metal affinities have been measured for multiple metals, the metal preferences of bacterial metal sensor proteins again tend to simply abide by the Irving-Williams series (78, 79, 82).

### Affinity, Access (Kinetics), and Allostery

A series of publications in the first decade of this century revealed that metal specificity of metal sensors can be determined by three factors. First, metal affinity contributes toward metal selectivity. Second, the allosteric mechanism connecting metal binding to altered DNA binding or to gene activation can respond selectively to different metals. Finally, the kinetics of access can differ for different sensors, for example due to delivery proteins (10, 82).

### Relative Affinity, Access and Allostery

Since 2010 it has become evident that affinity, allostery, and access operate as relative parameters in a set of sensors (83–85). Such observations are now possible because sufficiently large numbers of bacterial metal sensors have been characterized. Metal selectivity is now seen to result from the concerted actions of a cell's complement of metal sensors. In this manner, specificity is not constrained by absolute metal preferences (10, 82). The best sensor in the set is the sensor that responds. What defines the best in the set for each metal?

**FIGURE 3.**
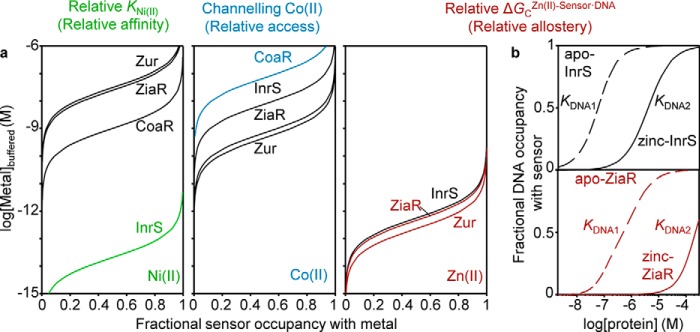
**Relative affinity, relative access, and relative allostery in a complement of metal sensors influences the metals detected *in vivo*.** *a*, calculated fractional occupancy of InrS, Zur, ZiaR, and CoaR with Ni(II), Zn(II), and Co(II) as the concentration of these elements changes: Fractional occupancy: θ= [Metal]_buffered_/(K_Metal_ + [Metal]_buffered_) using published *K*_Metal_ (83–85). *b*, fractional occupancy of specific DNA (*top*) with apo- (*dashed*) and zinc-InrS (*solid*) and (*bottom*), apo- (*dashed*) and zinc-ZiaR (*solid*), as a function of protein concentration. Coupling free energy: Δ*G*_C_ = −*RT*ln(*K*_DNA2_/*K*_DNA1_). The simulated curves were generated using published *K*_DNA_ values (85), [DNA] = 10 nm. The selective detection of nickel correlates with relative nickel affinity, of zinc with relative Δ*G*_C_ for zinc, but a major kinetic contribution (channeling) is invoked for cobalt.

Recent studies of the metal sensors of the model organism *Synechocystis* PCC 6803 exemplify the contributions of relative affinity, relative allostery, and relative access. By examining one sensor from each family of metal sensors present in this organism, the parameter correlating with selective metal detection was found to vary from metal to metal (Fig. 3). Importantly, the absolute metal preferences, as reflected in *K*_Metal_ values of InrS (nickel-responsive efflux derepressor), CoaR (cobalt-responsive efflux activator), and ZiaR and Zur (zinc-responsive efflux derepressor and influx corepressor, respectively) (61, 83, 86, 87), do not universally match their metal specificities *in vivo*. Rather, the detection of nickel correlates with relative nickel affinity, and the detection of zinc correlates with relative free energy coupling DNA binding to zinc binding (relative allostery), but a substantial kinetic contribution is invoked in the selective detection of cobalt (relative access) (83–85) (Fig. 3).

To elaborate, InrS possesses the tightest nickel affinity in this set of metal sensors (83). Thus, as the buffered concentration of nickel rises, provided the distribution of nickel among the sensors approximates to the thermodynamic equilibrium state, InrS will trigger nickel efflux before the concentration becomes sufficiently high for nickel to aberrantly bind to any of the other sensors (Fig. 3*a*) (83). This assumes roughly equivalent numbers of molecules of each sensor per cell (a parameter that in the future needs to be measured). Cognizant of the challenges in determining protein-metal affinities and noting the weak *K*_Ni(II)_ of ZiaR and Zur, a series of interprotein competition experiments also confirmed that nickel partitions from each of the other sensors to InrS (83).

In contrast to nickel, cobalt affinities do not correlate with *in vivo* specificities; rather, cobalt-sensing CoaR has the weakest *K*_Co(II)_ of the set of sensors (84), (Fig. 3*a*). Moreover, *in vitro*, cobalt promotes DNA association by Zur and DNA dissociation by ZiaR, yet neither ZiaR nor Zur responds to cobalt *in vivo* under conditions in which CoaR responds (84). This implies that cobalt is channeled to CoaR and away from ZiaR and Zur with their tighter cobalt affinities. There is evidence that CoaR is membrane-associated, and cobalt acquisition may involve channeling via the cobalamin biosynthetic complex, which is also membrane-associated. Additionally, there is evidence that CoaR may not solely sense cobalt directly, but also detect an intermediate in the B_12_ assembly pathway (84). In summary, CoaR has preferential access to the cobalt effector relative to ZiaR and Zur.

The zinc affinity of InrS is comparable with the sensory sites of ZiaR and Zur (Fig. 3*a*), yet following prolonged zinc exposure, ZiaR responds but InrS does not. Critically, although the allosteric mechanism of InrS is capable of responding to zinc, the coupling free energy linking zinc binding to DNA binding (Δ*G*_C_^zinc·sensor·DNA^) is greater for ZiaR than for InrS (85), (Fig. 3*b*). In short, zinc is a more effective derepressor of ZiaR than of InrS. Thus, at some equivalent fractional zinc occupancies, a greater proportion of InrS relative to ZiaR will be bound to DNA. InrS can thereby repress its gene target, whereas the ZiaR target remains derepressed. This exemplifies how relative coupling free energy Δ*G*_C_, that is relative allosteric effectiveness, in a complement of metal sensors can also dictate selectivity (Fig. 3*b*).

## Improbable Kinetics and Associative Metallation

Metal affinities of metal sensors for the most competitive metals such as nickel, zinc, and copper are so tight that it is not credible for metal partitioning to and from solution to reach equilibrium in a viable timeframe. The off-rates are too slow. However, this assumes dissociative metal exchange. As an alternative, associative metal exchange can occur to/from labile metal sites of proteins (including metal sensors) and components of a polydisperse buffer. This ill-defined buffer is composed of small molecules such as amino acids, glutathione, organic acids, and inorganic ligands, plus weak adventitious ligands on the surface of macromolecules, specific buffering proteins, and a subset of the delivery proteins. Rates of metal exchange in cells can thus be unexpectedly fast, and can swiftly approach the equilibrium state. Moreover, such a process of associative ligand exchange through a polydisperse buffer can operate at buffered concentrations below 10^−9^ m, the theoretical threshold for one atom per cell volume in a bacterium such as *E. coli* (88).

**FIGURE 4.**
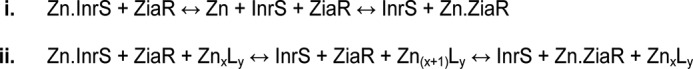
**Associative ligand exchange with a polydisperse buffer. i,** the transfer of zinc from InrS to ZiaR via a dissociative release of zinc from InrS to a hydrated state. **ii,** the transfer of zinc from InrS to ZiaR by (potentially swift) associative ligand exchange via a partly (*x*) zinc-saturated number of ligands (*y*) of a polydisperse buffer (*L*).

For the most competitive metals, the fully hydrated pool is indeed estimated to be below 10^−9^ m and thus equates to less than one (free) atom per cell at any instant (88, 89) (Fig. 2). In relation to Fig. 3 and the example in the preceding section, InrS does transiently respond to zinc *in vivo*, whereas the response of ZiaR is persistent. The buffered concentration of zinc would have to fall below 10^−11^ m for a protein with the *K*_Zn(II)_ of InrS to have less than full zinc occupancy to restore repression. Under these conditions, persistent ZiaR must therefore detect a pool of exchangeable zinc that is buffered at least 2 orders of magnitude below ∼10^−9^ m (85). One explanation is that ZiaR is metallated through associative ligand exchange with a polydisperse buffer rather than depending upon a hydrated pool of zinc ions. By way of illustration, the equations in Fig. 4 represent the transfer of zinc from InrS to ZiaR (**i**) by a dissociative process requiring the slow release of zinc from InrS to the hydrated state, and (**ii**) by potentially swift associative exchange with ligands of a buffer.

## Prospective: The Elements of Biotechnology and Biomedicine

With such a large proportion of enzymes requiring metals, discord between their metal binding preferences and metal requirements has implications for biological chemistry, as well as applications in biomedicine and biotechnology. For example, knowledge of the *in vivo* metallation states of components of metabolic and signaling networks is required to improve the accuracy of systems biology computations. Synthetic biology aims to engineer cells for new purposes. Success may often depend upon an ability to coincidentally rewire the circuitry for enzyme metallation.

## Footnotes

2 The abbreviations used are:

ARD: acireductone dioxygenase
SOD: superoxide dismutase
CCS: copper chaperone for superoxide dismutase.
